# Uninformative polymorphisms bias genome scans for signatures of selection

**DOI:** 10.1186/1471-2148-12-94

**Published:** 2012-06-22

**Authors:** Marius Roesti, Walter Salzburger, Daniel Berner

**Affiliations:** 1 Zoological Institute, University of Basel, Vesalgasse 1, Basel, CH-4051, Switzerland

**Keywords:** Allele frequency distribution, F_ST_, Gasterosteus aculeatus, Genetic marker, Hitchhiking, Population differentiation, Singleton

## Abstract

**Background:**

With the establishment of high-throughput sequencing technologies and new methods for rapid and extensive single nucleotide (SNP) discovery, marker-based genome scans in search of signatures of divergent selection between populations occupying ecologically distinct environments are becoming increasingly popular.

**Methods and Results:**

On the basis of genome-wide SNP marker data generated by RAD sequencing of lake and stream stickleback populations, we show that the outcome of such studies can be systematically biased if markers with a low minor allele frequency are included in the analysis. The reason is that these ‘uninformative’ polymorphisms lack the adequate potential to capture signatures of drift and hitchhiking, the focal processes in ecological genome scans. Bias associated with uninformative polymorphisms is not eliminated by just avoiding technical artifacts in the data (PCR and sequencing errors), as a high proportion of SNPs with a low minor allele frequency is a general biological feature of natural populations.

**Conclusions:**

We suggest that uninformative markers should be excluded from genome scans based on empirical criteria derived from careful inspection of the data, and that these criteria should be reported explicitly. Together, this should increase the quality and comparability of genome scans, and hence promote our understanding of the processes driving genomic differentiation.

## Background

A major challenge in evolutionary biology is to understand how natural selection acts on molecular genetic variation [1-4]. One approach to studying the consequences of selection at the genomic level is the application of genome scans that screen a collection of polymorphic genetic marker loci for their extent of differentiation between multiple (typically two) populations occupying ecologically distinct environments. Loci or genomic regions displaying particularly high population differentiation (usually quantified by an F_ST_ estimator [5]) relative to some differentiation baseline (reflecting primarily neutral drift) are interpreted as either being directly under divergent selection, or exhibiting genetic hitchhiking along with a quantitative trait locus (QTL) under divergent selection [6-9]. Genome scans therefore have the potential to illuminate the link between ecological selection and molecular variation, and hence to contribute to our understanding of adaptive diversification. This is particularly true if information from genome scans is integrated with complementary lines of evidence such as QTL mapping [10].

Genome scans can be performed in different ways, depending on the genomic resources available for a focal research system. On the one hand, reference-free (anonymous) scans are carried out without information on the physical genomic position of a marker locus. Here the F_ST_ value for each locus is treated as an independent data point and is evaluated against a baseline distribution derived from the entire data set *e.g.*, [11-14]. Loci exhibiting extreme F_ST_ values relative to the baseline (‘outlier loci’) are then interpreted as being directly or indirectly influenced by divergent selection. (Note that we here use divergent selection in a broad sense, including situations where an allele is selected in one environment but neutral in the other.) On the other hand, reference-based genome scans map loci physically to an available genome *e.g.*, [15-18]. This offers a great advantage: loci occurring in the same genomic neighbourhood, and consequently exhibiting some physical linkage, will tend to display correlated F_ST_ values that can be integrated by taking a sliding window approach. This allows not only the identification of genomic regions displaying exceptionally high population differentiation, but also exploring the number and physical extent of such regions [3]. Moreover, depending on the marker resolution, outlier regions may be screened for candidate genes potentially targeted by divergent selection.

Inferences drawn from both reference-free and reference-based genome scans obviously depend on the availability of reliable polymorphism data. The objective of our study is to highlight a potential problem with polymorphism data sets that can introduce bias to genome scans and lead to incorrect interpretations of genomic differentiation, or the lack thereof. The problem lies in F_ST_ being sensitive not only to the extent of genetic differentiation among populations, but also to the allele frequency distribution. Specifically, very low F_ST_ values (*i.e.*, near zero, or even negative values, depending on the formula used for calculation) at a polymorphic marker locus can arise for two different reasons: first, when the locus’ polymorphism involves alleles segregating at relatively even frequencies in both populations, but the frequency distribution of the alleles does not differ between the populations (upper example in Table 1). For such a locus, inferring the absence of population differentiation would generally be reasonable.

**Table 1:** Differentiation between two populations, as quantified by Weir and Cockerham’s F_ST_ estimator theta [[19]]

	**Genotypes population A**			**Genotypes population B**			**F**_**ST**_
Informative polymorphism	TT	TC	CC	TT	TC	CC	
	5	10	5	5	10	5	-0.026
Uninformative polymorphism	TT	TC	CC	TT	TC	CC	
	20	0	0	19	1	0	0.000

Other F_ST_ estimators produce qualitatively similar results), given informative and uninformative single nucleotide polymorphism at a marker locus (two alleles are present, T and C).

Second, a very low (or negative) F_ST_ value will also arise if the alleles at a marker locus exhibit an extremely skewed frequency distribution. That is, if a locus is nearly monomorphic in both populations but contains an alternative allele segregating at very low frequency such that this allele occurs only once or a few times in the entire data set (lower example in Table 1). Such a locus is *constrained* to display a very low F_ST_ value between the populations [11]. However, inferring the absence of population differentiation from this F_ST_ value is problematic. The reason is that such rare alleles primarily represent relatively recent mutations, most of which will experience rapid stochastic loss [20]. Markers with a very low minor allele frequency therefore lack the adequate sensitivity to capture the historical signatures of drift and hitchhiking, the key processes in genome scans.

**Figure 1:**
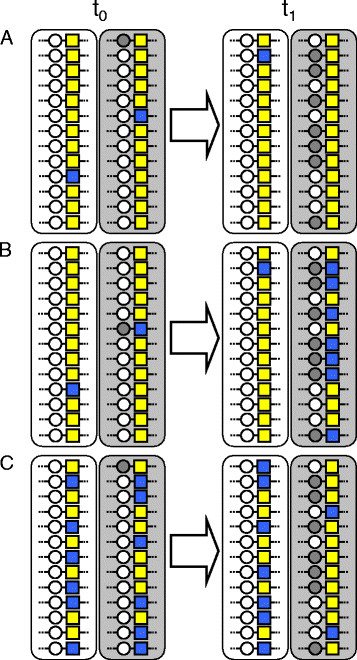
**Informative and uninformative markers in genome scans.** Two populations derived from the same ancestral population occupy ecologically distinct environments (white and gray boxes) at t_0_,. Circles represent an ecologically important QTL with two alleles under divergent selection; white and gray alleles are favored in the white and gray environment. Squares represent a neutral marker with two alleles (yellow and blue). The marker is tightly physically linked to the QTL. In **A**), both initial (t_0_) populations display a very low frequency for the blue marker allele. A novel adaptive QTL allele arising in the gray habitat will therefore likely be associated with the frequent yellow marker allele. When sampling the populations at t_1_, after a period of selection that has increased the frequency of the gray QTL allele in the gray environment, no signature of selection is visible at the marker locus because hitchhiking along with the favored QTL allele has not materially changed the allele frequency distribution at the marker (F_ST_[22] approximates zero at both t_0_ and t_1_). In **B**), the initial conditions (t_0_) are as in A), except that the novel adaptive QTL allele happens to be linked to the rare blue marker allele. At t_1_, selection at the QTL will be visible at the marker (F_ST_ = 0.22) because the blue allele has hitchhiked to high frequency. In **C**), the initial (t_0_) allele frequency distribution at the marker is relatively even in both populations (F_ST_ = 0). At t_1_, the marker exhibits a clear signature of selection (F_ST_ = 0.13) because the yellow allele has increased in frequency by hitchhiking. In both B) and C) but not in A), we would consider the marker locus informative at t_1_ based on its minor allele frequency across both samples, and consider the marker for a genome scan for the signature of selection (see text).

To illustrate this point, imagine that a novel QTL allele arises in the neighborhood of a nearly monomorphic marker. This QTL allele is unlikely to be linked to the rare allele at the marker. If the QTL allele is favored by selection and increases in frequency within the population where it arose, hitchhiking along with the QTL will produce only a very minor (if any) allele frequency shift at the marker locus (Figure 1A). Population differentiation at the QTL will therefore not be visible at the linked marker. A clear signature of hitchhiking, however, will be seen if the marker displays a more balanced allele frequency distribution (Figure 1C; or if the QTL allele happens to be linked to the rare marker allele, Figure 1B). A similar inconsistency in differentiation between selected QTL and associated markers with highly skewed allele frequency distribution also occurs in the situation where selection acts on standing variation (soft sweep; [21]).

Of course, in addition to the situation where a *natural* allele segregates at very low frequency within populations, a highly skewed allele frequency distribution at a locus can also arise artificially during marker data acquisition. For instance due to PCR replication or sequencing error. The locus then produces a minimal F_ST_ value although correctly no F_ST_ value would be calculated because the locus is not polymorphic. However, many strategies exist to avoid such technical errors (including achieving high sequencing coverage, or re-sequencing; see also [23] and references therein). Our paper is therefore primarily concerned with biological polymorphisms.

To summarize, there are two fundamentally different causes for minimal F_ST_ values in genome scan data sets: polymorphisms with relatively even allele frequency distribution, but without population differentiation, *versus* polymorphisms with extremely skewed allele frequency distribution unable to pick up population differentiation. Hereafter, we refer to these forms of polymorphisms as ‘informative’ *versus* ‘uninformative’. We emphasize, however, that we restrict this crude classification to genome scans searching for signatures of selection in the form of elevated differentiation. Markers with highly skewed allele frequency distributions might well be informative in other analytical contexts, such as the estimation of mutational or demographic parameters based on allele frequency spectra [24,25].

If uninformative polymorphisms are abundant in a marker data set used for a genome scan (and they generally will, see below), we can predict a number of undesirable consequences: in both reference-free and reference-based approaches, the estimated overall baseline differentiation, which is considered to reflect the effect of drift, will be biased downward. As a consequence of this bias in the baseline, the number of loci considered outliers driven by divergent selection in reference-free genome scans may be inflated. By contrast, in sliding window type of scans, the magnitude of among-population differentiation in genomic regions influenced by selection will be weakened, or in the worst case erased. Both effects can lead to incorrect conclusions about the genomic consequences of divergent selection. We emphasize that these problems will arise irrespective of the specific estimator used to quantify population differentiation, or the method chosen for outlier detection. That is, uninformative marker loci will also influence sophisticated methods that estimate F_ST_ for a locus by taking into account genome-wide differentiation and locus-specific sample size [14], or approaches based on P-values from locus-specific significance tests (*e.g.*, [16]).

It would thus seem straightforward to eliminate uninformative marker loci from polymorphism data sets prior to performing a genome scan, as reflected in Beaumont and Nichols’ [11] recommendation to preferably use loci with high heterozygosity for such analyses. However, a screen of 24 recent genome scan papers based on single nucleotide polymorphisms (SNPs), including most such studies currently available, suggests that the above issue is not generally recognized. (Note that our paper focuses on SNPs because this marker type is becoming standard in population genomics; but the conclusions hold for any type of marker.) Only three studies report marker filtering according to some minor allele frequency threshold ([18,26,27]; the latter study excluded singleton loci only, *i.e.*, markers with the minor allele occurring only a single time). It is therefore possible that patterns reported and conclusions drawn in many genome scan studies are unreliable to some extent. Given that genome scans are becoming increasingly easy to perform owing to the advent of high-throughput sequencing technology [28], new techniques for extensive SNP discovery (in particular restriction site associated DNA (RAD) sequencing [29]), and automated data analysis pipelines, the problem of bias arising from uninformative marker loci deserves wide recognition. A first goal of our study is therefore to use extensive SNP data from lake and stream population of stickleback fish to demonstrate that uninformative marker loci indeed have the potential to bias results from genome scans. Our second goal is to show that such bias can be avoided through careful inspection of the data set and subsequent exclusion of uninformative marker loci based on empirical criteria.

## Methods

Our study uses SNP data from threespine stickleback (*Gasterosteus aculeatus*) populations occurring in lake and stream habitats within two independently colonized drainages. The first is the Lake Constance drainage in Switzerland (the ‘COW’ lake-stream population pair from [30]), hereafter called the ‘Constance system’. The divergence between the lake and stream population in this system appears to be recent (a few hundred years). The second is the Boot Lake drainage on Vancouver Island, Canada (the Boot sites ‘L’ and ‘S2’ in [31]), hereafter called the ‘Boot system’. Lake-stream divergence in this system is more ancient (thousands of years). Lake and stream stickleback are known to experience divergent selection [31,32], and the specific population pairs were chosen because they differ in the magnitude of habitat-related phenotypic and neutral genetic (microsatellite) divergence (stronger divergence in the older Boot system than in the younger Constance system). For further details on the locations and populations see [30,31]. All samples were taken with permission from the British Columbia Ministry of Environment (permit number NA06-20791), and the fisheries authority of the canton Thurgau.

For SNP detection, we Illumina-sequenced RAD [29] derived from 27 stickleback specimens from each of the four sites (*i.e.*, one lake and one stream site in two drainages; total N = 108). Library preparation essentially followed the method described in [17]. In short, DNA was digested by using the *Sbf1* restriction enzyme and barcode-ligated for each individual separately. Amplified barcoded DNA was then single-end sequenced on an Illumina genome analyzer IIx with 76 cycles in libraries of 18 pooled individuals each. The Illumina short reads (sequenced RAD sites; deposited at the NCBI Short Read Archive, accession number SRP007695) were parsed by individual barcode, and for each individual separately aligned to the stickleback genome (Ensembl database version 63.1, assembly Broad S1) using Novoalign v2.07.06 (http://novocraft.com). Alignment to a unique genome position was enforced, effectively eliminating sequences derived from repeated elements. The average sequence coverage per individual and RAD site was 27 and 31 for the lake and stream sample in the Constance system, and 30 and 11 for the Boot system. Alignments were converted to BAM format using Samtools v0.1.11 [33]. For each individual and RAD site, we then determined the consensus diploid genotype if ten or more replicate reads were available, or a haploid consensus genotype if replication was below ten. This threshold was chosen because for polymorphic nucleotide positions, we identified heterozygote diploids based on a binomial test with insufficient power at low replication. This test involved calculating the binomial likelihood of the observed frequency distribution of the SNP alleles under the null hypothesis of heterozygosity (*i.e.*, assuming a probability of 0.5 for both alleles). Positions were considered heterozygous if the likelihood was greater than 0.01. Consensus genotyping was quality-aware in that bases with a greater than 0.01 calling error probability were excluded from the binomial test.

To find SNP markers and calculate genome-wide lake-stream population differentiation within each of the two systems, we pooled individual consensus genotypes from the lake and stream sample for each RAD site. If at least 27 genotypes were available from *each* of the two habitats, we proceeded with F_ST_ calculation. In other words, a RAD site was considered only if each individual contributed at least one haploid consensus genotype on average to the site’s genotype pool. For F_ST_ calculation, the genotype pool for each RAD site was screened base by base for polymorphisms. If a variable position occurred, we calculated F_ST_ based on haplotype diversity (equation 7 in [22]). For RAD sites exhibiting multiple SNPs, we retained only the highest F_ST_ value observed across all variable base positions. (Using the average F_ST_ value across all positions, or selecting a single SNP at random, produced very similar results supporting identical conclusions.) Negative F_ST_ values were rounded to zero, as commonly done.

The above F_ST_ calculation considered *any* type of SNPs. To explore the effect of informative *versus* uninformative markers, we repeated the above F_ST_ calculation protocol by imposing the restriction that the minor (less frequent) allele had to occur at least *n* times in the lake-stream genotype pool, where *n* spanned the range from two to ten in increments of one. (The above default F_ST_ calculation represents the case with *n* = 1.) For each calculation series, we then computed the number of resulting SNPs, and the mean F_ST_ value across all SNPs. We also visualized genomic differentiation by a sliding window approach using local polynomial fitting (LOESS) implemented in R (R Development Core Team [34]; 2^nd^ order polynomial with band width of 0.4; using simpler polynomials and different band widths did not alter our conclusions). All post-sequencing analysis except for alignment and file conversion was coded in the R language, making use of the Bioconductor packages ShortRead [35], Biostrings, and Rsamtools.

## Results

**Figure 2:**
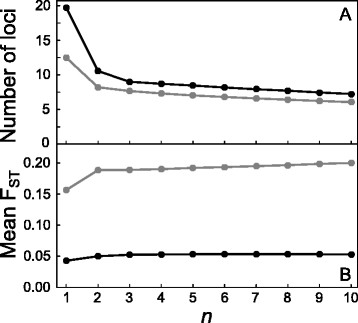
**The number of polymorphic loci (x 10**^**3**^**) (A), and mean F**_**ST**_** across all loci (B), for different minor allele count thresholds (*n*) in the Constance (black) and Boot (gray) lake-stream stickleback system.** This threshold specifies the minimum number of times the minor SNP allele at a locus had to occur in the pooled lake and stream sample for a polymorphic locus to remain in the data set.

In both the Constance and Boot stickleback population pair, raising the threshold for the minimal required count of the minor SNP allele (*n*) had a dramatic influence on the number of polymorphic marker loci available for F_ST_ calculation. Most strikingly, the number of SNPs dropped by 46.5% (from 19,729 to 10,546) and 34% (from 16,729 to 7,546) in the Constance and Boot system when singleton loci were excluded by setting *n* to two (Figure 2A). Increasing *n* from two to ten, however, had a relatively minor effect on the number of polymorphic loci. Our stickleback data sets thus exhibit a very high proportion of singleton loci, as generally found in empirical studies (*e.g.*, [36-39]). The genomic location of these singleton loci did not show any systematic association with chromosome position (details not).

**Figure 3:**
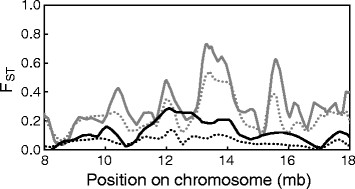
**Differentiation along a segment of chromosome seven between the lake and stream stickleback population from the Constance (black) and Boot (gray) system.** Sliding window analysis was performed by local polynomial fitting of F_ST_ values for data sets with the allele frequency threshold *n* set to one (*all* SNPs in the data sets considered; dotted lines), and *n* set to four (at least four copies of the minor allele required across the pooled lake and stream sample in each system; solid lines). Note the relatively flat differentiation profiles with *n* = 1.

Including these uninformative marker loci in the genome scan led to the consequences predicted above. First, baseline differentiation was substantially lower than the differentiation obtained when setting *n* to two or greater (Figure 2B). For instance, genome-wide F_ST_ increased by 17% and 20% in the Constance and Boot system when raising *n* from one to two. In absolute terms, this shift was more dramatic in the Boot system displaying the higher overall differentiation between the populations. Second, F_ST_ profiles obtained from sliding window analyses including all markers (*n* = 1) were strikingly flatter than those from analyses excluding uninformative polymorphisms. These two consequences are visualized for a segment of chromosome seven (Figure 3), which is representative of what we found throughout the genome. For that specific genomic region, analyses with and without uninformative marker loci might lead to qualitatively different conclusions about the magnitude and physical extent of population differentiation. For example, in the Constance system, a large segment ranging approximately from 12–14 mb displays elevated differentiation, as revealed when using informative markers only. This differentiation is certainly substantial, given the low baseline differentiation in that young system (Figure 2B), and might indicate ongoing divergent selection in that genomic region. Nevertheless, elevated differentiation within that region would probably not be recognized when tolerating uninformative markers in the sliding window analysis.

Note that in Figure 3, we define informative marker loci as those with the minor allele occurring at least four times (*n* = 4), resulting in an average inter-locus distance of 53 kb and 63 kb for the Constance and Boot system. This minor allele threshold eliminated bias associated with uninformative marker loci relatively effectively; choosing higher thresholds had a relatively minor influence on the sliding window profiles.

## Discussion

Our empirical analysis demonstrates that abundant uninformative polymorphisms in a genome scan data set can bias the estimated baseline differentiation, and hence affect conclusions about the genomic signatures of selection.

In our stickleback data set, uninformative polymorphisms (essentially in the form of singleton loci) were very abundant. Illumina sequencing type one errors (*i.e.*, wrong base calls despite high indicated base call quality) in RAD sequences poorly replicated at the individual level might contribute to this pattern [23,39]. To examine this possibility, we inspected 50 randomly chosen SNPs exhibiting zero F_ST_ from the full data set accepting any type of polymorphisms (*i.e.*, minor allele count threshold *n* = 1) for each lake-stream system. As expected, a high proportion of these markers were singleton loci (Constance: 28 [56%]; Boot: 35 [70%]). For the Boot system with lower replication per locus in the stream sample (see above), 15 of the 35 singleton loci represented unreplicated RAD sequences. For these loci, the minor allele is likely a sequencing error.

However, all but one of the singleton alleles in the Constance system represented consensus genotypes integrating multiple (2–26, mean: 9.1) replicate RAD sequences. Hence, the bulk of the uninformative marker loci in our data clearly *cannot* be attributed to sequencing error, because the probability of multiple identical errors at a specific nucleotide position at a given RAD site is practically zero. The abundance of rare SNP alleles therefore represents a real biological feature of the studied stickleback populations (acknowledging a small potential contribution from PCR artefacts). This is not unexpected: theory consistently predicts a skew toward polymorphisms with low minor allele frequency, and hence a high proportion of singleton polymorphisms, under a broad range of demographic and selective conditions [24,36,40-44]. Bias associated with uninformative polymorphisms is therefore of general importance to genome scan studies, and not specific to our empirical system. Our analysis also raises a caveat regarding marker densities; the effective number of markers providing relevant information in genome scans might often be dramatically lower than the number reported.

In the present study, excluding singleton polymorphisms had the greatest influence on the results. Reliable quantification of differentiation patterns, however, might require substantially more stringent minor allele frequency thresholds. (Note that such marker filtering also effectively eliminates any sequencing and PCR error from the data.) Bradbury *et al.*[27], for instance, excluded SNPs exhibiting an overall minor allele frequency of 0.25 or less, and a similar threshold was adopted in a recent lake-stream stickleback study carried out in our lab [45]. To obtain a guideline for marker filtering, the latter RAD-based study evaluated the strength of the correlation in F_ST_ values between ‘sister’ RAD sites (*i.e.*, DNA sequences flanking the *same* restriction site in the genome) in relation to increasingly stringent minor allele frequency thresholds (see Appendix S2 in the Supporting information to [45]). The rationale was that if an F_ST_ value provided by a given marker reliably quantifies the consequences of drift and selection in a genomic region, then another extremely tightly linked marker should yield a similar F_ST_ value. This approach, however, requires tightly physically linked markers data and substantial population differentiation (otherwise the correlation in F_ST_ between linked will remain poor even with stringent marker filtering).

## Conclusions

Given the rapidly increasing feasibility and popularity of genome scans for signatures of selection, researchers should be aware that uninformative polymorphisms need to be excluded from data sets. This is not achieved by just avoiding technical errors, as a high prevalence of nearly monomorphic loci is a general biological feature of samples from natural populations. We suggest that a reasonable strategy to define and eliminate uninformative polymorphisms should be chosen by inspecting the allele frequency distribution of the polymorphisms, and by assessing the influence of different marker filtering thresholds on the genomic patterns of interest, or appropriate statistics (such as the correlation of F_ST_ between sister RAD sites). Also, the approach taken to eliminate uninformative polymorphisms should be reported explicitly. Together, this should increase the quality and comparability of genome scans, and hence promote our understanding of the processes shaping genomic differentiation.

## Competing interest

The authors declare no competing interest.

## Authors’ contributions

DB and MR conceived the study; WS provided materials and infrastructure; MR and DB generated the sequence data; DB and MR analyzed the data; DB wrote the paper, with input from MR and WS. All authors read and approved the final manuscript.
